# Normal values for sagittal spinal alignment: a study of Brazilian subjects

**DOI:** 10.6061/clinics/2018/e647

**Published:** 2018-11-23

**Authors:** Raphael R Pratali, Mohamed A Nasreddine, Bassel Diebo, Carlos Eduardo A.S. Oliveira, Virginie Lafage

**Affiliations:** I Departamento de Ortopedia e Traumatologia, Hospital do Servidor Publico Estadual de Sao Paulo, Sao Paulo, SP, BR; II Department of Orthopaedic Surgery, State University of New York (SUNY) Downstate Medical Center, Brooklyn, NY, USA; III Department of Ortho Surgery, Hospital for Special Surgery, New York, NY, USA

**Keywords:** Spinal Curvature, Bone Alignment, X-rays, Epidemiologic Measurements, Reference Values

## Abstract

**OBJECTIVES:**

The purpose of this study is to investigate the normal values of and chain of correlations between spinopelvic parameters in a Brazilian population.

**METHODS:**

This is a prospective observational study including asymptomatic adult subjects who had full spinal radiographs performed. The subjects were stratified by age into 3 groups (18-39 years old, 40-59 years old, and >60 years old), and radiographic parameters were compared across age groups and gender using ANOVA and Student's *t*-test, respectively. The relationships between various radiographic parameters were evaluated with Pearson correlation coefficients.

**RESULTS:**

One hundred and thirty asymptomatic volunteers (mean age, 48 years) met the inclusion criteria. The mean sagittal parameters in a normal Brazilian population were as follows: lumbar lordosis (LL) of 56.8°, pelvic tilt (PT) of 12.4°, pelvic incidence (PI) of 49.4°, PI–LL of -7.4°, T1 pelvic angle (TPA) of 8°, sagittal vertical axis (SVA) of -0.54 cm and T1 slope of 25.2°. Subjects ≥60 years old had significantly higher values of SVA (*p*=0.024) and TPA (*p*=0.009) than the two younger age groups. The TPA was significantly correlated with the following spinopelvic parameters: LL (r=-0.172, *p*=0.005), PT (r=0.776, *p*<0.001), PI (r=0.508, *p*<0.001), PI–LL (r=0.717, *p*<0.001), SVA (r=0.409, *p*<0.001) and T1 slope (r=0.172, *p*=0.050).

**CONCLUSION:**

This study demonstrated significant physiologic trunk inclination with increasing age. The TPA, an angular parameter of global spinal alignment, presented a chain of correlations with different spinal segments.

## INTRODUCTION

The sagittal alignment of the human spine is characterized by curvatures that can be measured by radiographic parameters and classified by morphology 1. Appreciating the variations among individuals may help to elucidate the relationship between spinal alignment and the development of spinal pathologies including adult spinal deformity (ASD). Sagittal alignment in asymptomatic subjects with an upright posture is comprised of a chain of correlations from the pelvis to the cervical spine 2. The shape of the pelvis is correlated with the curvature of the lumbar spine, which in turn is correlated with the curvature of the thoracic spine, and this chain of correlations continues through the cervical spine 2. As described by Dubousset, this chain of correlations positions the center of gravity of the trunk over the pelvis, which is key to maintaining an economic posture 3.

In the setting of ASD, recent studies have demonstrated that spinopelvic alignment is correlated with patient-reported outcomes, specifically pain and disability 4-6. This finding was the premise of the recent Scoliosis Research Society (SRS)-Schwab classification of ASD, in which sagittal modifiers (sagittal vertical axis (SVA), pelvic tilt (PT), and pelvic incidence (PI) minus lumbar lordosis (LL)) capture the clinically relevant descriptors of spinal deformity 7,8. Recently, the T1 pelvic angle (TPA) was introduced as the angle extending from a line from the femoral head to the center of the T1 vertebral body and a line from the femoral head to the center of the superior sacral endplate. The TPA has been shown to be related to both PT and SVA and is correlated with health-related quality of life in patients with ASD 9,10.

The vast majority of spinopelvic parameters considered for the development of the SRS-Schwab classification and the TPA are based solely on a homogeneous North American cohort and did not take into account diversity in age, ethnicity, geographic locale, etc., seen in the ASD patient population. In addition, significant differences in pelvic and spinopelvic alignment have been reported across different ethnicities 11,14; thus, it is valuable to investigate this variability in spinal radiographic parameters. If differences do exist, surgeons can then customize surgical objectives for treating deformities in specific patient populations. The aims of the present study were 1 to determine the normal values of and related chains of correlations between spinopelvic parameters in a Brazilian population and 2 to analyze the effect of the age on these parameters.

## MATERIALS AND METHODS

### Design, setting, participants and ethics

This is an observational, prospective cohort study involving asymptomatic adult volunteers in a convenience sample analysis. Institutional review board approval was obtained prior to the initiation of data collection. For study enrollment, 148 asymptomatic volunteers had their radiographic images evaluated from March 2014 through June 2015. Written consent was obtained from all study subjects. Adult subjects older than 18 years were included in this analysis. Subjects were excluded if they had any neck or back pain, previous spinal or neurological surgery, underlying neurological or neuromuscular conditions, history of spinal trauma or neoplastic disease and/or complaint of hip/knee/ankle/foot disability that could potentially alter the ability to take accurate standing radiographs. A total of 18 volunteers were excluded from the study because of poor quality radiographs that did not allow adequate visualization of the entire spine and/or both femoral heads. All volunteers included in the analysis were inhabitants of Sao Paulo, although 39% of the patients were born in other states (Minas Gerais and different states in the northeast region).

### Variables and measurements

**Figure 1:**
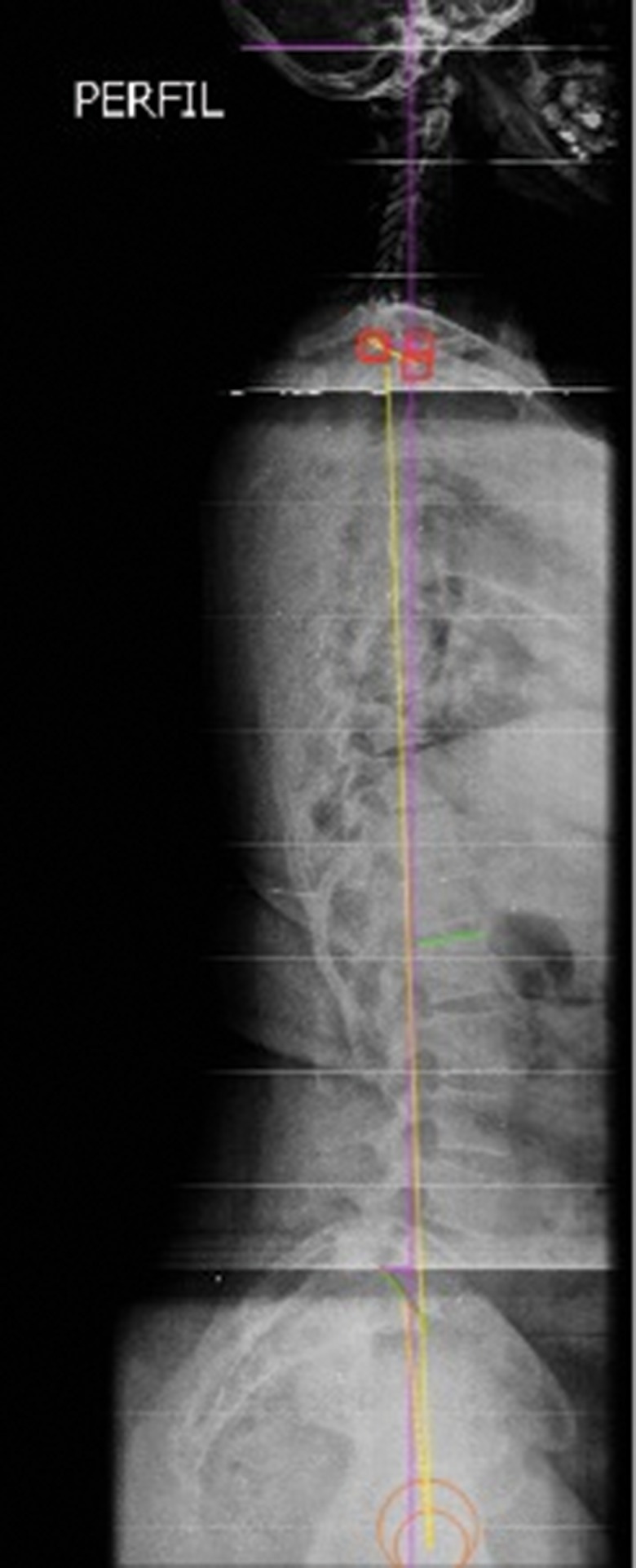
Example of measurement of the radiographic parameters using Surgimap software (Nemaris Inc. New York, USA).

Data collection at baseline included demographic and radiographic information. Radiograph acquisitions followed a strict protocol: coronal and sagittal full spinal radiographs were obtained with patients standing in a comfortable standing posture with their fingers on their clavicles or on their face and their shoulders at a 45° forward elevation 4,15. Radiographic parameters were measured using Surgimap software (Nemaris Inc., New York, USA), a validated 16 tool for spinal analysis (Figure 1). The parameters included were: L1-S1 LL 5,6, PI 3,5,6, PT 3,5,6, sacral slope (SS) 3,5,6, SVA 4,5,6, T1 slope 2 and TPA 9,10. The difference between PI minus LL (PI–LL) was also calculated 6. All measurements were performed twice by the same observer, and verified by a second more experienced observer. Discrepancies greater than 10% were reviewed and reevaluated if necessary.

### Statistical analysis

Statistical analysis was performed using SPSS, version 20.0. The normality of the data was investigated using the Kolmogorov-Smirnov test. The subjects were stratified by age into 3 groups: 18-39 years old, 40-59 years old, and >60 years old. The mean, standard deviation, and range for each parameter in each group were calculated. Radiographic parameters for different gender groups were compared using Student's *t*-test; radiographic parameters by age group were investigated using ANOVA. Pearson product-moment correlation coefficients were calculated to identify the relationships between various radiographic parameters and were subsequently presented on a scatter-plot with TPA and each of the other radiographic parameters. A significance level of *p*<0.05 was adopted.

## RESULTS

### Population data

A total of 130 asymptomatic volunteers met the inclusion criteria and were evaluated. The mean age of the cohort was 48.15 years (SD=±15.3; range, 18-78 years). Eighty-one patients (62.3%) were women, and 49 (37.7%) were men. The total sample was divided into three age groups: 44 volunteers (33.85%) in the 18-39 years group, 44 volunteers (33.85%) in the 40-59 years group, and 42 (32.3%) in the ≥60 years group.

### Radiographic values

The mean values of the radiographic parameters analyzed in the total sample are summarized in Table 1. Analysis of the mean values of these parameters revealed large variability in sagittal profiles among the volunteers. However, considering the radiographic criteria for spine deformity used by the International Spine Study Group (coronal Cobb angle=20 degrees, SVA=5 cm, PT=25°, and/or thoracic kyphosis=60°) 7,9, only four volunteers in the sample (3%) had findings consistent with spinal deformity including one patient with a PT=25 degrees and three with an SVA≥5 cm (5.0 cm, 5.01 cm and 6.39 cm).

Radiographic comparisons of sagittal alignment by gender (Table 2) revealed that female subjects had a significantly higher mean LL (58.3±7.3°) than male subjects (54.2±8.5°) and a significantly lower mean PI–LL (-8.7±7.3°) than male subjects (-5.1±8.0°), *p*<0.05, with no significant differences in PI or other radiographic parameters.

Analysis by age group (Table 3) demonstrated a significantly higher mean SVA and TPA in the subjects ≥60 years old than in those in the two other age groups (*p*=0.024 and *p*=0.009). There were no other significant differences discovered among the three age groups for the remaining radiographic parameters.

### Correlation of the T1 pelvic angle with other radiographic parameters

**Figure 2:**
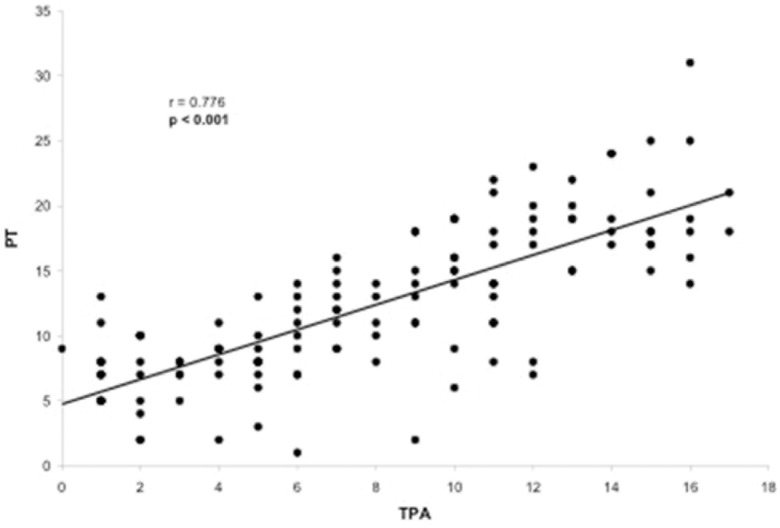
Scatter-plot of the regression analysis presenting a significant and substantial positive correlation between the T1 pelvic angle and pelvic tilt.

**Figure 3:**
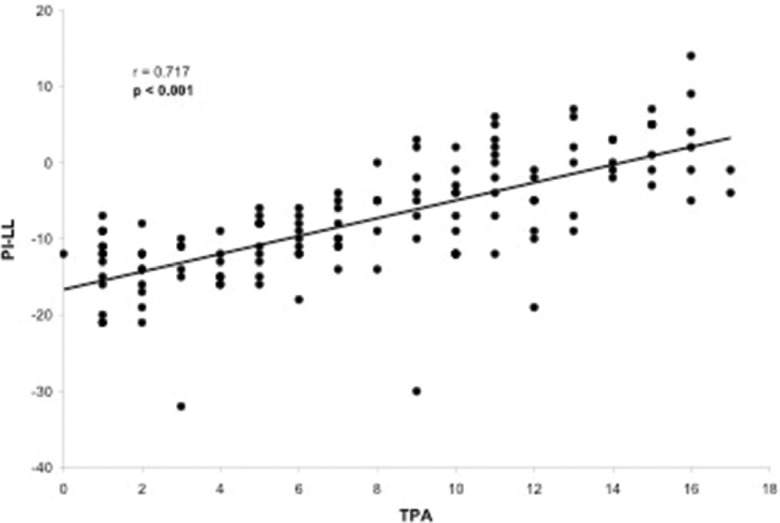
Scatter-plot of the regression analysis presenting a significant and substantial positive correlation between the T1 pelvic angle and the difference in pelvic incidence and lumbar lordosis.

**Figure 4:**
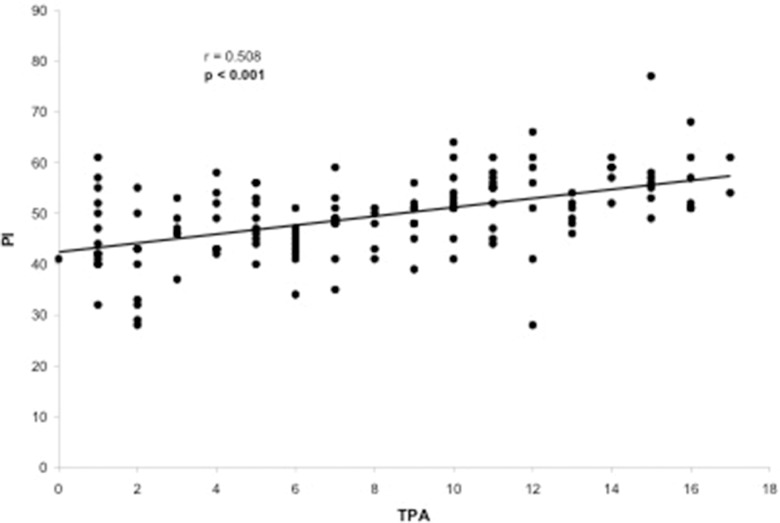
Scatter-plot of the regression analysis presenting a significant and reasonable positive correlation between the T1 pelvic angle and pelvic incidence.

**Figure 5:**
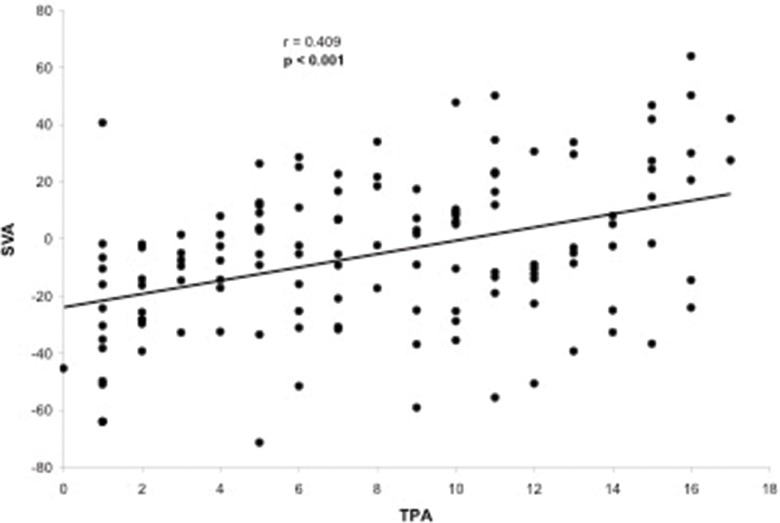
Scatter-plot of the regression analysis presenting a significant positive and reasonable correlation between the T1 pelvic angle and sagittal vertical axis.

**Figure 6:**
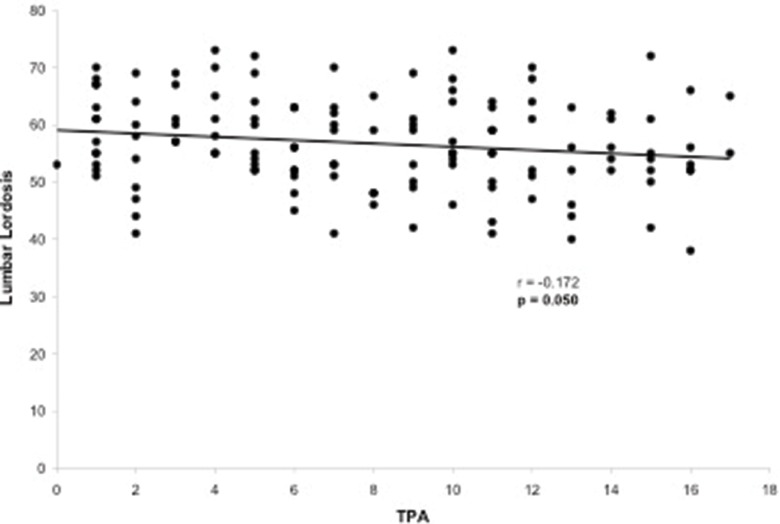
Scatter-plot of the regression analysis presenting a significant but weak negative correlation between the T1 pelvic angle and lumbar lordosis.

**Figure 7:**
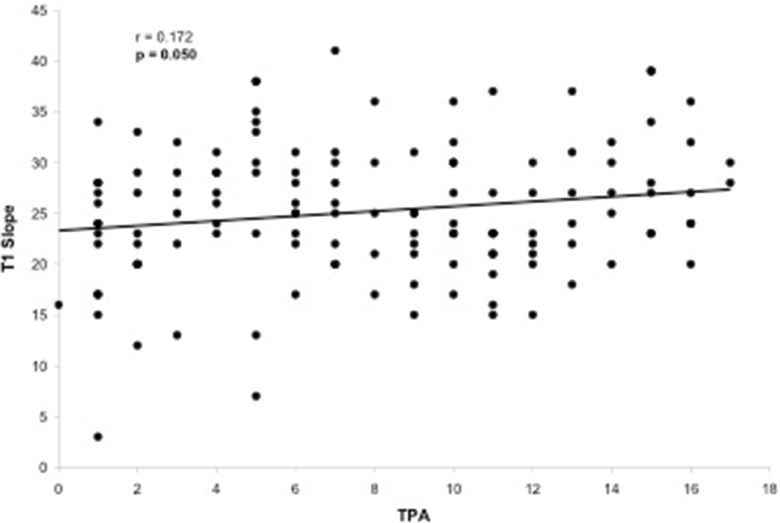
Scatter-plot of the regression analysis presenting a significant but weak positive correlation between the T1 pelvic angle and T1 slope.

Evaluation of the correlation between the TPA and other radiographic parameters demonstrated a significant correlation between the TPA and the following parameters: LL (*p*=0.005), PT (*p*<0.001), PI (*p*<0.001), PI–LL (*p*<0.001), SVA (*p*<0.001) and T1 slope (*p*=0.050). The only radiographic parameter that was not correlated with the TPA was the SS (*p*=0.677). The TPA presented a substantial correlation with PT (r=0.776, Figure 2) and PI–LL (r=0.717, Figure 3) and a reasonable correlation with PI (r=0.508, Figure 4) and SVA (r=0.409, Figure 5). Despite of statistical significance, the correlations between the TPA and LL (r=-0.172, Figure 6) and between the TPA and the T1 slope (r=0.172, Figure 7) were weak.

## DISCUSSION

The interest in ASD has been growing over the past few years. This growing interest is in accordance with the demographic shift observed in most countries around the world, with the number of elderly individuals rising to unprecedented levels. Recent studies have revealed that as many as 60% of individuals older than 60 years have some degree of ASD, 17 and the correlation between radiographic parameters and disability has consistently been proven by validated health measures 4-6,18,.

Defining normal values for radiographic parameters is important for understanding ASD and for establishing radiographic thresholds in the setting of sagittal realignment surgery. Several reports investigating normal values across different populations have demonstrated a lack of uniformity 11-14. However, these publications have major limitations, the most significant being the lack of standardization in the enrollment period and methods of evaluating sagittal alignment parameters. To date, there is no information on normal spinopelvic alignment values in the Brazilian population, and these data are presented in the present study.

The Brazilian population studied presented with variable LL (min. of 38° and max. of 73°). Female volunteers had significantly higher values of LL than did the male volunteers, in accordance with the findings in the work by Vialle et al. 13. Considering the clinical importance of the relationship between PI and LL, expressed as PI–LL 6, the data from the present study demonstrate a significantly lower mean difference between PI and LL in female volunteers than in male volunteers. As PI did not differ between genders, a lower mean value of PI–LL in females was expected since they also had a higher mean LL. Furthermore, the value of PI–LL in the study population was within 10° in both females and males, which is in accordance with the findings of previous literature 6.

The significantly higher mean SVA in the group with older subjects (≥60 years old) is consistent with the results demonstrated by Mendoza-Lattes et al. 20. In both studies, the increase in SVA was not accompanied by a significant decrease in the mean LL. Only three asymptomatic volunteers presented with an SVA≥5 cm, satisfying the radiographic criterion for spinal deformity defined by the International Spine Study Group 7. One patient was 23 years old, and the other two were ≥60 years old. Forward truncal inclination was observed with increasing age in our sample, including in asymptomatic volunteers, and should be considered a physiological change in sagittal spinal alignment resulting from the normal aging process.

Recently, Protopsaltis et al. 9,10 introduced the TPA, an angular measure of global sagittal spinal deformity established in the setting of ASD. In the present study, normal values of the TPA in asymptomatic Brazilian volunteers are reported, along with comparisons of the TPA among individuals of different genders and ages. To date, this is the first analysis of normal values of the TPA in asymptomatic subjects. The mean TPA was not significantly different in female and male subjects but was significantly higher in the group of older subjects (≥60 years old) than in the other age groups. Considering the threshold suggested for the TPA 9, no volunteers presented with a value ≥20°, corresponding to the cut-off of severe disability (Oswestry Disability Index (ODI)>40) 21. However, 20 asymptomatic volunteers (15.3%) presented with a value ≥14°, which is considered to be the cut-off for minimal disability (ODI>20) 21. Of these volunteers with a TPA≥14°, 20% (4/20) were between 18-39 years old, 30% (6/20) were between 40-59 years old and 50% (10/20) were ≥60 years old. Since the TPA is the sum of the T1 spinopelvic inclination and PT, and the mean PT was not different between the three age groups, the increase in TPA suggests a tendency of the trunk to incline anteriorly with increasing age. This finding is similar to the significant increase in SVA that was noted with increasing age.

The TPA has been shown to be correlated with other radiographic parameters including SVA, PT, PI–LL, and T1 spinopelvic inclination 9. In the present study, the TPA was also correlated with all the spinopelvic alignment radiographic parameters that were analyzed (SVA, PI, PT, LL, PI-LL and T1 slope) but not with the SS. More specifically, we observed high correlations between the TPA and PT (r=0.776) and between the TPA and PI–LL (r=0.717). Lee et al. 22 reported that the T1 slope, which is a component of the thoracic inlet angle, determines the amount of subaxial cervical lordosis required to maintain the head in a balanced position and that it varies depending on global spinal alignment as measured by the SVA. In our sample, a significant, but weak, correlation between the TPA and the T1 slope was observed, which contributes to both pelvic and global spinal alignment. This finding may support the existence of a link between cervical and spinopelvic sagittal alignment and the concept that the spinal regions are not independent of one another 2,23.

Recently, age-related changes in alignment and patient-reported outcomes have been discussed in the literature. Schwab et al. reported an analysis of the relationship between objective parameters, such as age and radiographic alignment parameters (PI–LL, PT, SVA, and TPA), and patient-reported outcomes, such as the ODI and the Short-Form (SF)-36 Physical Component Score (PCS). The study indicated that radiographic alignment thresholds were higher in older populations than in younger populations and that interpretation of radiographic alignment should be corrected for age 24. Our data revealed a significant increase in anterior truncal inclination (SVA and TPA) with increasing age in a sample of asymptomatic subjects; this increase should not be considered ASD but is due to the normal aging process of the spine.

The main limitation of the present study is that it involved a homogeneous sample of volunteers who were inhabitants of just one region of the country (Sao Paulo). Since Brazil has a continental territorial extension and a heterogeneous population, it may not represent the total population. However, the data presented are important as current reference values. Further multicentric multiregional studies are warranted to address an actual representative population database.

The analysis of spinopelvic alignment in a Brazilian sample of asymptomatic volunteers revealed a significantly higher SVA and TPA and demonstrated physiological trunk inclination with increasing age. The TPA, a novel angular parameter of global spinal alignment, presented a chain of correlations with different spinal segments.

**Conflicts of Interest.** The author(s) declared the following potential conflicts of interest with respect to the research, authorship, and/or publication of this article: Virginie Lafage has received grants as part of speaking/teaching arrangements with NuVasive, K2M, MSD, and DePuy Spine, is a consultant for NuVasive, has received research support from NuVasive, DePuy Spine, K2M, Stryker (paid through ISSGF) and SRS, has stock options from Nemaris Inc., and is a board member/committee member of SRS (research grant & 3D task force) unrelated to the submitted work. The remaining authors have no conflicts of interest to report.

## AUTHOR CONTRIBUTIONS

Pratali RR was responsible for the conception and design, statistical analysis and manuscript drafting. Nasreddine MA was responsible for the acquisition of data. Pratali RR, Diebo B and Lafage V were responsible for the analysis and interpretation of data and critical revision of the manuscript. All of the authors participated in the review of the submitted version and approved the final version of the manuscript.

## Figures and Tables

**Table 1:** Radiographic parameters of the entire cohort. There was great variability in the parameters among the volunteers.

VARIABLE	MIN	MAX	MEDIAN	MEAN (SD)
LUMBAR LORDOSIS (LL)	38°	73°	56°	56.8° (±8°)
PELVIC TILT (PT)	1°	31°	11°	12.4° (±5.8°)
PELVIC INCIDENCE (PI)	28°	77°	50°	49.4° (±8.2°)
PI–LL	-32°	14°	-8°	-7.4° (±7.7°)
SACRAL SLOPE (SS)	21°	60°	37°	37.2° (±6.7°)
T1 PELVIC ANGLE (TPA)	0°	17°	8°	8° (±4.7°)
SAGITTAL VERTICAL AXIS (SVA)	-7.13 cm	6.39 cm	-0.54 cm	-0.54 cm (±2.7 cm)
T1 SLOPE	3°	41°	25°	25.2° (±6.6°)

**Table 2:** Radiographic parameters by gender. There were significant differences between male and female volunteers in lumbar lordosis and the difference in pelvic incidence and lumbar lordosis.

VARIABLE	GENDER		*p*
	Male (N=49) Mean (SD)	Female (N=81) Mean (SD)		
LUMBAR LORDOSIS (LL)	54.2° (±8.5°)	58.3° (±7.3°)	**0.004**
PELVIC TILT (PT)	11.6° (±5.6°)	12.8° (±6°)	0.261
PELVIC INCIDENCE (PI)	49.1° (±6.8°)	49.6° (±9°)	0.732
PI–LL	-5.1° (±8°)	-8.7° (±7.3°)	**0.010**
SACRAL SLOPE (SS)	37.7° (±6.1°)	36.8° (±7.1°)	0.496
T1 PELVIC ANGLE (TPA)	8.1° (±4.5°)	7.8° (±4.9°)	0.743
SAGITTAL VERTICAL AXIS (SVA)	-0.11 cm (±2.98 cm)	-0.8 cm (±2.49 cm)	0.157
T1 SLOPE	26° (±7.1°)	24.7° (±6.2°)	0.276

Student’s *t*-Test.

**Table 3:** Radiographic parameters by age group. There were significant differences in the T1 pelvic angle and sagittal vertical axis among the different age groups.

VARIABLE	AGE GROUP			*p*
	18-39 years (N=44) Mean (SD)	40-59 years (N=45) Mean (SD)	≥60 years (N=41) Mean (SD)		
LUMBAR LORDOSIS (LL)	55.3° (±8.8°)	57.8° (±°8.8)	57.2° (±5.8°)	0.291
PELVIC TILT (PT)	11.5° (±5.5°)	11.8° (±6.6°)	13.8° (±5°)	0.151
PELVIC INCIDENCE (PI)	48.6° (±7.9°)	48.8° (±9.6°)	50.9° (±6.6°)	0.367
PI–LL	-6.6° (±7°)	-9° (±8.3°)	-6.2° (±7.7°)	0.186
SACRAL SLOPE (SS)	36.9° (±6.6°)	37.2° (±8.2°)	37.4° (±5.1°)	0.948
T1 PELVIC ANGLE (TPA)	7.2° (±4.4°)	7° (±4.8°)	9.8° (±4.5°)	0.009
SAGITTAL VERTICAL AXIS (SVA)	-6.7 cm (±2.9 cm)	-1,22 cm (±2.52 cm)	0.35 cm (±2.47 cm)	0.024
T1 SLOPE	25.4° (±7.9°)	24.4° (±6.2°)	25.9° (±5.5°)	0.588

ANOVA.
